# eIF6 is potential diagnostic and prognostic biomarker that associated with ^18^F-FDG PET/CT features and immune signatures in esophageal carcinoma

**DOI:** 10.1186/s12967-022-03503-7

**Published:** 2022-07-06

**Authors:** Yan Gao, Lingling Yuan, Jing Zeng, Fuyan Li, Xiaohui Li, Fan Tan, Xusheng Liu, Huabing Wan, Xueyan Kui, Xiaoyu Liu, Changbin Ke, Zhijun Pei

**Affiliations:** 1 Department of Nuclear Medicine and Institute of Anesthesiology and Pain, and Department of Pathology, Taihe Hospital, Hubei University of Medicine, No. 32, Renmin Road, Shiyan, 442000 China; 2 Hubei Key Laboratory of Embryonic Stem Cell Research, Shiyan, 442000 China

**Keywords:** eIF6, Esophageal carcinoma, ^18^F-FDG-PET parameter, GLUT1, Immune signatures, Prognostic biomarker

## Abstract

**Background:**

Although eukaryotic initiation factor 6 (eIF6) is a novel therapeutic target, data on its importance in the development of esophageal carcinoma (ESCA) remains limited. This study evaluated the correlation between eIF6 expression and metabolic analysis using fluorine-18 fluorodeoxyglucose (^18^F-FDG) -Positron emission tomography (PET) and immune gene signatures in ESCA.

**Methods:**

This study employed The Cancer Genome Atlas (TCGA) to analyze the expression and prognostic value of eIF6, as well as its relationship with the immune gene signatures in ESCA patients. The qRT-PCR and Western blot analyses were used to profile the expression of eIF6 in ESCA tissues and different ESCA cell lines. The expression of tumor eIF6 and glucose transporter 1 (GLUT1) was examined using immunohistochemical tools in fifty-two ESCA patients undergoing routine ^18^F-FDG PET/CT before surgery. In addition, the cellular responses to eIF6 knockdown in human ESCA cells were assessed via the MTS, *EdU*, flow cytometry and wound healing assays.

**Results:**

Our data demonstrated that compared with the normal esophageal tissues, eIF6 expression was upregulated in ESCA tumor tissues and showed a high diagnostic value with an area under curve of 0.825 for predicting ESCA. High eIF6 expression was significantly correlated with shorter overall survival of patients with esophagus adenocarcinoma (p = 0.038), but not in squamous cell carcinoma of the esophagus (p = 0.078). In addition, tumor eIF6 was significantly associated with ^18^F-FDG PET/CT parameters: maximal and mean standardized uptake values (SUVmax and SUVmean) and total lesion glycolysis (TLG) (rho = 0.458, 0.460, and 0.300, respectively, p < 0.01) as well as GLUT1 expression (rho = 0.453, p < 0.001). A SUVmax cutoff of 18.2 led to prediction of tumor eIF6 expression with an accuracy of 0.755. Functional analysis studies demonstrated that knockdown of eIF6 inhibited ESCA cell growth and migration, and fueled cell apoptosis. Moreover, the Bulk RNA gene analysis revealed a significant inverse association between eIF6 and the tumor-infiltrating immune cells (macrophages, T cells, or Th1 cells) and immunomodulators in the ESCA microenvironment.

**Conclusion:**

Our study suggested that eIF6 might serve as a potential prognostic biomarker associated with metabolic variability and immune gene signatures in ESCA tumor microenvironment.

## Introduction

Esophageal carcinoma (ESCA) is a common type of malignant tumors, which is divided into two main subtypes: esophageal squamous cell carcinoma (ESCC) which accounts for 90% of the ESCA, while the remaining is esophageal adenocarcinoma (EA) [1]. Due to lack of specific treatment for ESCC and methods for its early diagnosis, five-year survival rate remain below 15% [2]. ^18^F-fludeoxyglucose positron emission tomography/computed tomography (^18^F-FDG PET/CT) is a noninvasive and preferred early diagnostic tool, which is widely employed in the assessment of response to therapy, by simultaneous assessment of tumor morphology and metabolism over time [3]. Tumor FDG uptake is determined by the expression of glucose transporter family (GLUT1, 3 and 4) and glycolytic enzymes [4]. The PET-related parameters include the maximal and mean standardized uptake values (SUVmax and SUVmean), metabolic tumor volume (MTV), and total lesion glycolysis (TLG), which correlates with biological factors in the tumor microenvironment (TME) [5, 6]. Significant correlation was found between PET imaging parameters and the in vivo biological characterization of cancer lesions [7–9]. Our previous data also demonstrated that ^18^F-FDG PET/CT parameters have important clinical value in predicting novel molecular and clinical phenotypes of malignant tumors. The phenotypes include nucleophosmin 1 (NPM1) expression in lung cancer [10], eukaryotic translation initiation factor 2 Subunit β (EIF2S2) expression in colorectal cancer [11], and methyltransferase 3 (METTL3) expression in ESCA [12].

Eukaryotic initiation factor 6 (eIF6) is the first eIF associated with large 60S subunit in the nucleus, which is involved in translation initiation [13–15]. Previous studies demonstrated that eIF6 is highly expressed in human cancers such as colorectal cancer, hepatocellular carcinoma, ovarian serous carcinoma, acute promyelocytic leukemia, non-small cell lung cancer, and affect lymphoma genesis and tumor progression [16–20]. In addition, eIF6 translational activity regulate fatty acid synthesis and glycolysis through upregulation of lipogenic and glycolytic enzymes [21]. Scagliola et al. [22] showed that eIF6 induces a metabolic rewiring during progression from non-alcoholic fatty liver to hepatocellular carcinoma. However, the functions and molecular mechanisms of eIF6 in ESCA remain unclear. FDG uptake reflects glucose metabolism in malignant cells [6]. This study evaluated the relationship between eIF6 expression and glucose metabolism using FDG-PET and examined the prognostic significance in ESCA patients.

Both metabolic and immune-mediated syndrome in the TME have been associated with malignant tumor progression and metastasis [23, 24]. Recent studies have shown that there is overexpression of eIF6 mRNA in activated T cells and lymphoid cells [16, 25]. Besides, eIF6 was shown to be essential in overall immune system, particularly for metabolic switch in CD4^+^ T cell activation [26]. On the other hand, potential of immune checkpoint inhibition (ICI), such as blockage of programmed cell death-1 (PD-1)/ PD-1 ligand 1 (PD-L1) pathway, has been established in several tumors [27, 28]. However, this treatment option has not been applied to all patients, thus the need to find new targets.

In this study, we correlated the eIF6 expression with patient survival using public database [29], as well as ^18^F-FDG PET parameters in ESCA patients. We then used ESCA cell lines to elucidate the functions of eIF6. In addition, we attempted to investigate the association between the eIF6 expression and gene signatures of immune cells in ESCA. Our data showed that eIF6 might be a potential diagnostic and prognostic biomarker in ESCA patients.

## Materials and methods

### Patient samples

The study respectively reviewed fifty-five patients who were surgically treated and had pathologically confirmed ESCC in Taihe Hospital from January 2018 to July 2020. This study included patients who underwent ^18^F-FDG PET/CT imaging and were analyzed using immunohistochemistry (IHC) tests. The patients had available clinicopathological data, and none of them received chemo- or radiotherapy prior to the ^18^F-FDG PET/CT imaging. In this study, we used thirteen paired surgically obtained samples from patients with ESCA. The fresh-frozen ESCA tissues and matched non-tumor tissues were analyzed by qRT-PCR and Western blot.

### ^18^F-FDG PET/CT imaging and data analysis

Glucose metabolism was assessed by ^18^F-FDG PET/CT imaging using the Biograph mCT (64) system (Siemens Healthcare, Germany). As described [10, 12], the patients fasted for at least 6 h but had free access to water until the start of the imaging process. Whole-body position was monitored 50 min after intravenous administration of FDG (3.7–4.1 MBq/kg) and lasted for about 15 min. The PET/CT images were acquired following the manufacturer’s protocol. The metabolic parameters of the ^18^F-FDG PET images were evaluated by two experienced nuclear medicine physicians who were blinded to the final clinical diagnosis. Briefly, a region of interest (ROI) was placed around the primary tumor, and then FDG uptake in the lesions was determined from the PET images to calculate the maximum and mean SUV (SUV_max_ and SUV_mean_). SUV is defined as tissue concentration (Bq/g) × lean body mass (g)/injected dose (MBq)). A SUV_max_ threshold of 2.5 was used to define the metabolic tumor volume (MTV) (cm^3^), while total lesion glycolysis (TLG) is obtained by multiplying SUV_mean_ by MTV (TLG = ΣMTV × SUV_mean_) [30, 31]. In addition, the MTV and TLG were defined semi-automatically using an SUV-based platform as previously described [12].

### Quantitative real time PCR

Total RNA was isolated from frozen tissues and cell lines using TRIzol reagent (Thermo Fisher Scientific, USA). The RNA was used to synthesize cDNA with RT Master Mix kit (TaKara, China). The qRT-PCR experiment was performed using a TB Green Premix Ex Taq Kit (TaKaRa, China) in the Applied Biosystems ViiA TM 7 Real-time PCR system (Life Technologies, CA). We used β-actin as an internal control for normalization. The following primers were used:

- eIF6, Forward: 5′-GGCCGACCAGGTGCTAGTAGG-3′; Reverse: 5′- CACAC-CAGTCATTCACCACCATCC-3′.
- β-actin, Forward: 5′-TCTTCCAGCCTTCCTTCCT-3′; Reverse: 5′- AGCACTG-TGTTGGCGTACAG -3′.

### Western blot

As previously described [19], fresh tissues or cells were collected and digested with RIPA buffer containing 1% protease inhibitor on ice. The protein concentrations were measured by BCA method (Beyotime Biotechnology) and then resolved in SDS-PAGE gel. The protein samples were transferred onto PVDF membranes (Millipore, USA), followed by incubation with primary antibodies; anti-eIF6 (Thermofisher, Waltham, MA, USA), anti-GLUT1 (Abcam, Cambridge, UK), anti-Ecadherin (BD Pharmigen, USA), anti-Vimentin (Abcam), anti-Cytochrome c (Cyt c, Abcam), anti-CD45 (Proteintech, China), anti-CD11b (Abcam), anti-PD-L1 (Proteintech) or anti-GAPDH (Cell Signaling Technology, USA) at 4 °C overnight. The membranes were washed with TBST, and then incubated with secondary antibodies (Cell Signaling Technology, USA) for 1 h at room temperature. Finally, the proteins were visualized by an enhanced chemiluminescence (ECL) detection kit.

### Immunohistochemical (IHC) assay

IHC staining was performed following a previously described protocol [10]. Briefly, the samples were dissected on 5-μm-thick tissue sections embedded in paraffin, and then incubated with antibodies against eIF6 (1:400), GLUT1 (1:200), followed by secondary antibodies. Two pathologists who were blinded to clinical data independently analyzed the IHC data. The protein expression was profiled based on the staining intensity score, from 0 (negative), 1 + (weakly positive), 2 + (moderately positive) to 3 + (strongly positive). High- or low-expression was defined with scores equal to or above, and below the final IHC staining score 2, respectively.

### Gene expression pattern and patient prognosis in public datasets

We downloaded the mRNA expression (HTSeq counts) and associated clinical data for different human cancers from the TCGA (https://portal.gdc.cancer.gov/). The expression profiles were plotted in the ggplot2 R (https://github.com/tidyverse/ggplot2). On the other hand, survival curve was demonstrated using Kaplan–Meier plots [32]. To evaluate the predictive accuracy of eIF6 in TCGA cancers, “pROC” in R package was used to generate the time-dependent receiver operating characteristic (ROC) curve and then the area under curve (AUC) was calculated.

### Enrichment analysis and functional networks in ESCA

The eIF6 co-expression genes in the TCGA-ESCA were analyzed by DESeq2 R package. We then performed correlation analysis of the different variables using the Spearman’s or Pearson’s correlation test and Fisher’s exact test. Gene ontology (GO) biological process and Kyoto Encyclopedia of Genes and Genomes (KEGG) pathway enrichment analysis were performed using the clusterProfiler in R package (p.adj < 0.1 & qvalue < 0.2). Thereafter, top 200 protein coding genes significantly correlated with eIF6 were screened out for construction of PPI networks. Each network node was determined and visualized with CytoHubba plugin in Cytoscape. The top 10 genes with most connections were selected and considered to be ‘hub’ genes.

Next, the TCGA-ESCA samples were classified into two groups based on the eIF6A expression status. The edgeR package in Bioconductor was used to define differentially expressed genes (DEGs) between the eIF6 high and low ESCA samples (|log2FC|> 1.5, p value < 0.05). In addition, we used the GSEA to investigate meaningful biological processes. The hallmark gene sets and glycolysis signatures (REACTOME_GLYCOLYSIS, KEGG_GLYCOLYSIS_GKYCIBEIGENES, and HALLMARK_GLYCOLYSIS) were downloaded from the Molecular Signatures Database (MSigDB). The false discovery rate (FDR) cutoff of 0.25, and an adjusted p < 0.05 were considered significance. Besides, 282 glycolytic genes in three glycolysis signatures were downloaded from the MSigDB database. Pearson correlation coefficients of the eIF6 expression and glycolytic genes were calculated using TIMER database and DESeq2 R package in the TCGA database.

### Cell culture and transfection

Human epithelial cell line (HET1A cells) and ESCA cell lines (Eca109, KYSE30, KYSE150, CEC2) were obtained from the Cell Bank of Chinese Academy of Sciences (Shanghai, China). The cells were transfected with eIF6 siRNA using Lipofectamine 8000 transfection reagent (Beyotime, China), following the manufacturer’s instructions. The target sequences were: 5ʹ-CTGCTTTGCCAAGCTCACCAA-3ʹ for siRNA-1 (sieIF6-1) and 5ʹ-CTGGTGCATCCCAAGACTTCA-3ʹ for siRNA-2 (sieIF6-2).

### Cell viability analysis using MTS assay and *EdU* proliferation assay

The ESCA cells were seeded into 96-well plates and transfected with siRNA. After transfection for 0, 24, 48 or 72 h, we added MTS (CellTiter 96 Aqueous One Solution Cell Proliferation Assay, Promega) to each well and then incubated for 2 h. The optical density (OD) was then measured at 490 nm using a microplate reader (SpectraMax M3). Another proliferation assay was performed with the usage of an EdU kit (ClickTM, EDU 488, Beyotime) according to the manufacturers instruction. In brief, the transfected cells were incubated with diluted EdU (10 uM) for 2 h. Subsequently, cells were fixed and stained by Click Reaction Mixture and DAPI. Images were captured from five random fields under a fluorescent microscope. Finally, percentage of EdU positive cells was quantitated using Image J software.

### Flow cytometry analysis of apoptosis

Cell apoptosis was quantified by an Annexin V-FITC Apoptosis Detection Kit (BD Pharmigen, USA). The treated cells were harvested and washed twice with PBS. Afterwards, the cells were resuspended and then 3 µl FITC-conjugated annexin V and 10 µl propidium iodide staining solution were added. The rate of apoptosis was immediately measured using a FACS Calibur flow cytometer (BD Bioscience, USA).

### Wound healing assay

The cells were plated overnight and scratched by a 10 μL pipette tip. Subsequently, the cells were washed with sterile PBS and then refilled with complete medium. After transfection with siRNA, the wounded cells were analyzed every day by a microscope and then analyzed using Image J software.

### Estimation of immune cell characteristics

TCGA-ESCA patients were classified into low-expression or high-expression group based on the eIF6 expression. The xCell in R package was employed to evaluate immune and stroma cell abundance in the two groups. The relative immune signature score was estimated using the R GSVA package [33, 34]. In addition, the Tumor Immune System Interactions (TISIDB) database was used to study immune-related gens and immune subtypes, based on the eIF6 expression [35]. On the other hand, the Tumor Immune Estimation Resource (TIMER, https://cistrome.shinyapps.io/timer/) was used to define the correlation using the spearman correlation coefficient of a pair of genes and then estimated statistical significance, as well as tumor purity in ESCA. In addition, the correlation between the eIF6 expression and different immune signatures in ESCA samples in the GEPIA2 databases (http://gepia2.cancer-pku.cn) was calculated using Pearson’s correlation test.

### Statistical analysis

SPSS package (version 16.0, SPSS for Windows, 2007) and R package version (4.0.3) were used for statistical analyses. A p < 0.05 was used to define statistical significance. In this study, the survival and ROC analysis were carried out in R or corresponding R packages which included survival, survminer and pROC. For bivariate analysis, χ^2^ test, χ^2^ test or Fishers exact test were used. Spearman’s rho test was used to evaluate the correlation between the PET parameters and eIF6 expression levels in the ESCA patients. In addition, we used the greatest Youden index (sensitivity + specificity – 1) to define optimal cutoff value for the ROC curve. The T-test and Wilcox tests were used to compare differences between two groups, while comparisons among multiple groups were performed using the Tukey and the Wilcox tests. Statistical analysis and visualization were performed using the R Statistical Package and ggplot2 package.

## Results

### Upregulation of eIF6 is associated with ESCA patient prognosis

**Fig. 1:**
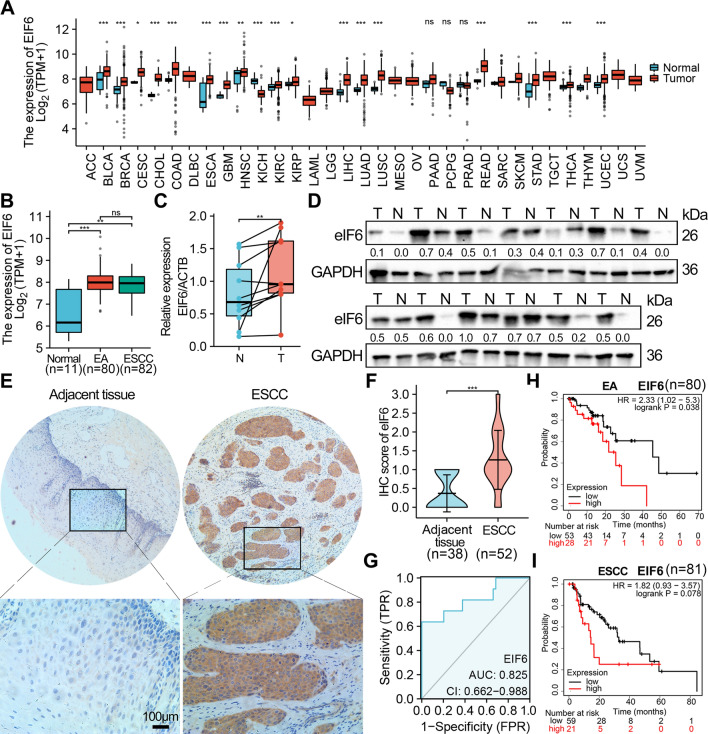
eIF6 is upregulated in ESCA tumor tissues and correlates with the prognosis of ESCA patients. **A** Expression patterns of human eIF6 mRNA across multiple types of tumor tissues and normal tissues. **B** eIF6 mRNA expression levels in EA (n = 80), ESCC tumor tissues (n = 82), and normal tissues (n = 11) from the TCGA-ESCA cohort. **C** The expression level of eIF6 was determined by qRT-PCR and Western blotting (**D**) in 13 paired ESCC tissues and adjacent tissues. T, ESCA tissues; N, paired adjacent normal tissues. **E** Representative IHC images and scores **F** of eIF6 in adjacent normal tissues and ESCC tissues (normal = 38, tumor = 52). **G** ROC curve showing the diagnostic value of eIF6 patients with ESCA (n = 162). **H** Kaplan–Meier curves showing the OS in EA and ESCC **I** patients based on the eIF6 mRNA expression level. *p < 0.05, **p < 0.01, ***p < 0.001

The eIF6 mRNA expression was analyzed in pan cancers in the TCGA database. The data demonstrated significantly higher expression of eIF6 mRNA in several cancer types, including bladder, breast, colorectal, ESCA, head and neck squamous cell, liver, lung, pancreatic, stomach, thyroid, and uterine corpus endometrial cancers, compared with normal tissues, while lower expression was observed in kidney cancer (Fig. 1A). In addition, a higher level of eIF6 expression was observed in ESCA and EA compared to normal tissues in the TCGA cohort (Fig. 1B). Thereafter, the eIF6 expression pattern was validated by qRT-PCR (Fig. 1C) and Western blot analyses (Fig. 1D) in fresh paired ESCC and adjacent patient tissues. Our data showed that eIF6 was significantly upregulated in ESCC tissues. We then performed IHC analysis in fifty-two patients and showed that the tumor tissues had higher expression of eIF6 compared with normal epithelia tissues (p < 0.05, Fig. 1E, F).

The 52 ESCC patients were divided into the eIF6-low (n = 35) and eIF6-high (n = 17) expression group. Subsequent analyses revealed that there was significant correlation between the eIF6 expression and SUV_max_ (p = 0.010) or SUVmean (p = 0.018), but not with gender, age, differential status, lymph node metastasis, p stage, TLG or MTV (Table 1). Finally, the ROC curve analyses demonstrated that eIF6 displayed superior diagnostic accuracy (AUC = 0.825, 95% CI, 0.662–0.988) which could distinguish the ESCA patients from healthy controls (Fig. 1G). Notably, higher eIF6 expression was negatively associated with survival time of patients with EA (n = 80, p = 0.038), but not with ESCC Kaplan-merrier plot (n = 81, p = 0.078) (Fig. 1H, I).

**Table 1:** Clinicopathological characteristics of 52 patients

Variables	eIF6—low (N, %)	eIF6—high (N, %)	*p*
Total	35(67.3%)	17 (32.7%)	
Clinical parameters			
Gender			1.000
Male	28 (53.8%)	14 (26.9%)	
Female	7 (13.5%)	3 (5.8%)	
Age ( years)			0.854
< 60	21 (40.4%)	9 (17.3%)	
≥ 60	14 (26.9%)	8 (15.4%)	
Differential			0.662
Poorly	14 (26.9%)	5 (9.6%)	
High/Moderately	21 (40.4%)	12 (23.1%)	
Lymph node metastasis			1.000
Negative	20 (38.5%)	9 (17.3%)	
Positive	15 (28.8%)	8 (15.4%)	
p Stage			0.098
1	12 (23.1%)	1 (1.9%)	
2	5 (9.6%)	4 (7.7%)	
3	18 (34.6%)	12 (23.1%)	
PET metabolic parameters	(median)		
SUV_max_			0.010*
Low	21 (40.4%)	3 (5.8%)	
High	14 (26.9%)	14 (26.9%)	
SUV_mean_			0.018*
Low	22 (42.3%)	4 (7.7%)	
High	13 (25%)	13 (25%)	
TLG			0.237
Low	20 (38.5%)	6 (11.5%)	
High	15 (28.8%)	11 (21.2%)	
MTV			1.000
Low	18 (34.6%)	8 (15.4%)	
High	17 (32.7%)	9 (17.3%)	

### Metabolic pathway enrichment analysis of eIF6 in ESCA

**Fig. 2:**
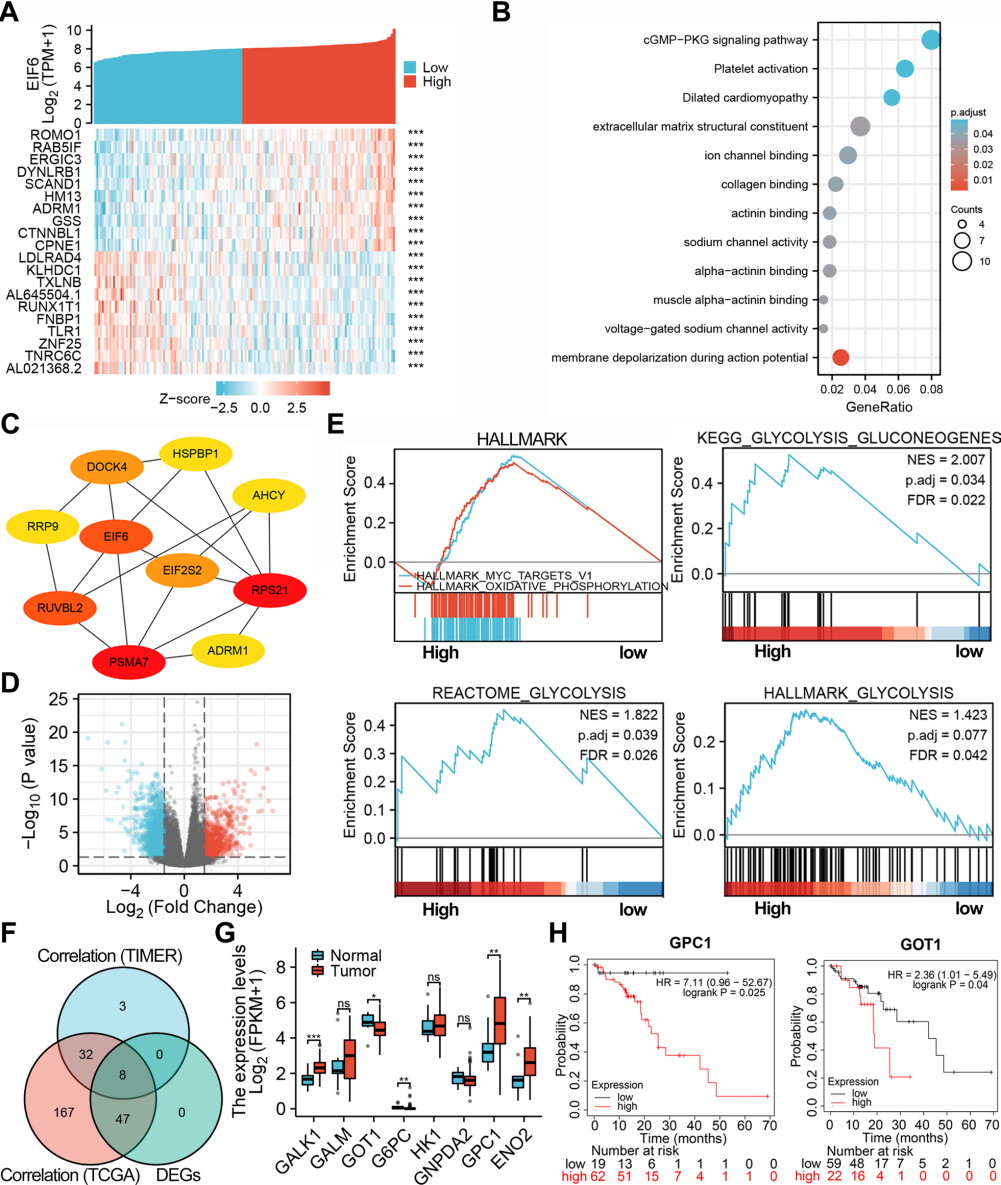
Functional analysis of the genes correlated or regulated by eIF6 in TCGA database. **A** A heat map of the top 10 genes correlated with eIF6 in the TCGA-ESCA patients. **B** Bubble diagrams showing the enrichment results of the top 200 genes correlated with eIF6 in ESCA. **C** The top 10 hub genes with the most connected degrees determined using the cytoHubba plugin in Cytoscape. **D** Volcano plots displaying the differentially expressed genes correlated with eIF6. **E** The HALLMARK pathways and glycolysis signatures that were significantly in patients with high or low eIF6 expression as determined with GSEA. **F** Venn diagram of the glycolytic genes that significantly correlated with eIF6 in TIMER database and TCGA database, and differentially expressed in high and low eIF6 expressing ESCA groups. **G** The expression of eight glycolytic genes identified from the Venn diagram in tumor tissues and normal tissues in TCGA-ESCA database. *p < 0.05, **p < 0.01, ***p < 0.001. **H** Overall survival curve of patients with ESCC according to their GPC1 or GOT1 expression in Kaplan–Meier plot database

To evaluate the biological interaction network and related signaling pathways associated with eIF6, the top 10 genes most positively or negatively correlated with eIF6 were analyzed in the heat map (Fig. 2A). ESCA eIF6 expression was positively correlated with ROMO1 expression (r = 0.64, p = 3.53E−20), but negatively associated with AL021368.2 (r = − 0.50, p = 1.16E−11). Our enriched GO terms analysis demonstrated that the genes that encode proteins correlated with eIF6 might be participating in membrane depolarization during action potential, constitute the extracellular structural matrix, alpha-actin in binding, and ion channel binding processes. The KEGG enrichment analysis showed that eIF6 is involved in cGMP-PKG signaling and platelet activation pathways (Fig. 2B). In addition, to identify top hub genes, we constructed a protein–protein interaction (PPI) network and showed that RPS21 and PSMA7 play key roles (Fig. 2C). On the other hand, a total of 2814 DEGs were screened upon eIF6 expression in 181 ESCA patients (Fig. 2D). GSEA was used to perform hallmark analysis for eIF6 and showed that most significant pathways in high- eIF6 group included HALLMARK_MYC_TARGETS (NES = 3.05, p.adjust = 0.017) and HALLMARK_OXIDATIVE_PHOSPHORYLATION (NES = 2.97, p.adjust = 0.018) (Fig. 2E). In addition, the functional analyses showed that glycolytic pathways such as KEGG_GLYCOLYSIS_GKYCIBEIGENES and REACTOME_GLYCOLYSIS were associated with eIF6 expression (p.adjust < 0.05) (Fig. 2E). Moreover, we employed correlation analysis to screen glycolytic genes that are differentially expressed in eIF6 high and low expression groups, including GALK1, GOT1, GPC1, ENO2, GALM, G6PC, HK1, and GNPDA2 (Fig. 2F). We further profiled the transcriptional expression and survival of the 8 significant genes in TCGA ESCA samples. Both GPC1 and GOT1 genes were upregulated in tumor tissues, and were implicated in poor ESCA prognosis (Fig. 2G, H, p < 0.05).

### Overexpression of eIF6 is associated with glucose metabolism in ESCA patients based on.^18^F-FDG PET-CT imaging

**Fig. 3:**
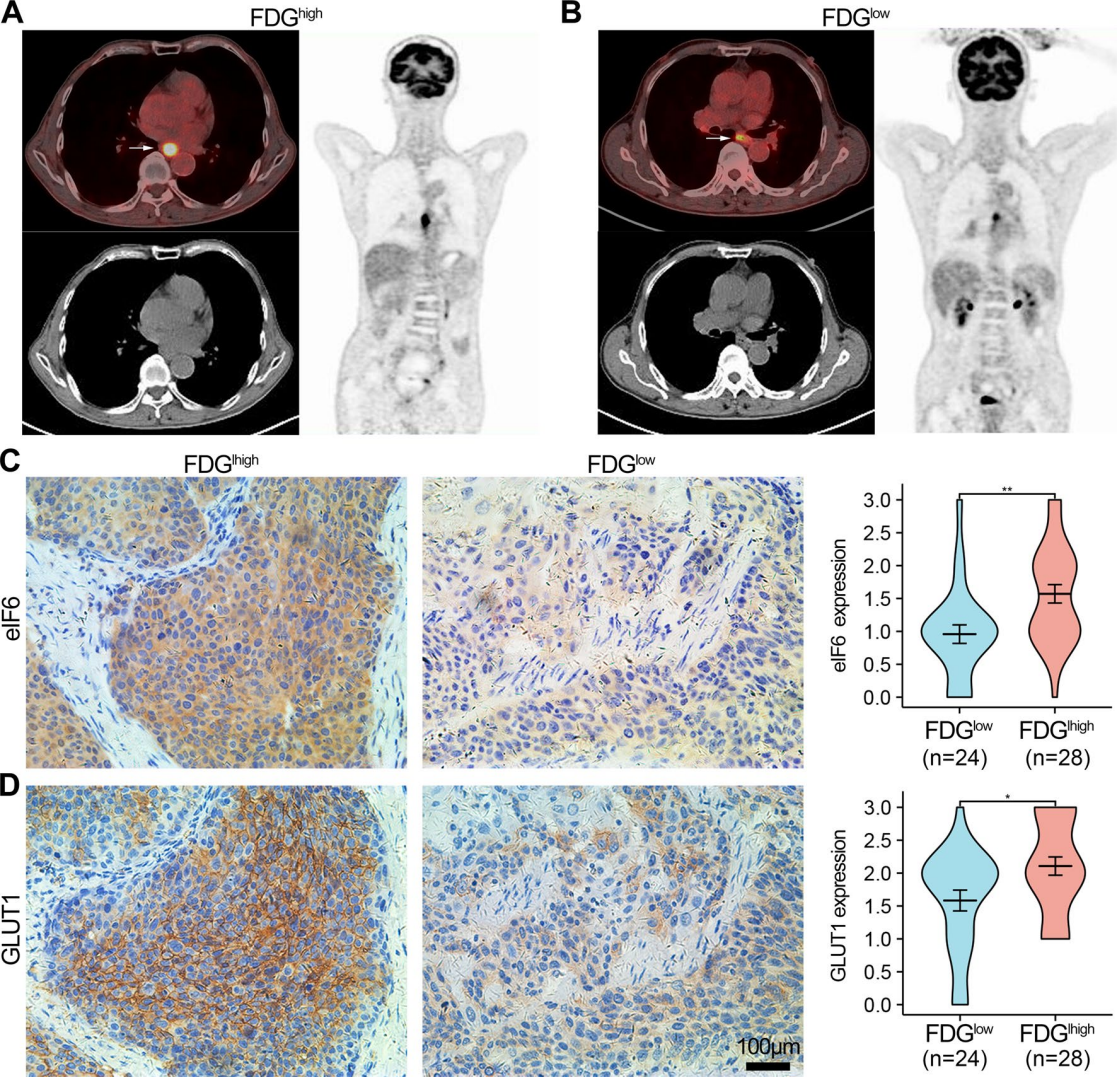
The expression of eIF6 and GLUT1 in ESCA patients with high and low ^18^F-FDG levels. **A**, **B** Representative PET/CT images for ESCC patients with different SUVmax values. **C** IHC staining for eIF6 and GLUT1 (**D**) in ESCC patients with high (n = 28) and low (n = 24) SUVmax values. *p < 0.05, **p < 0.01

**Fig. 4:**
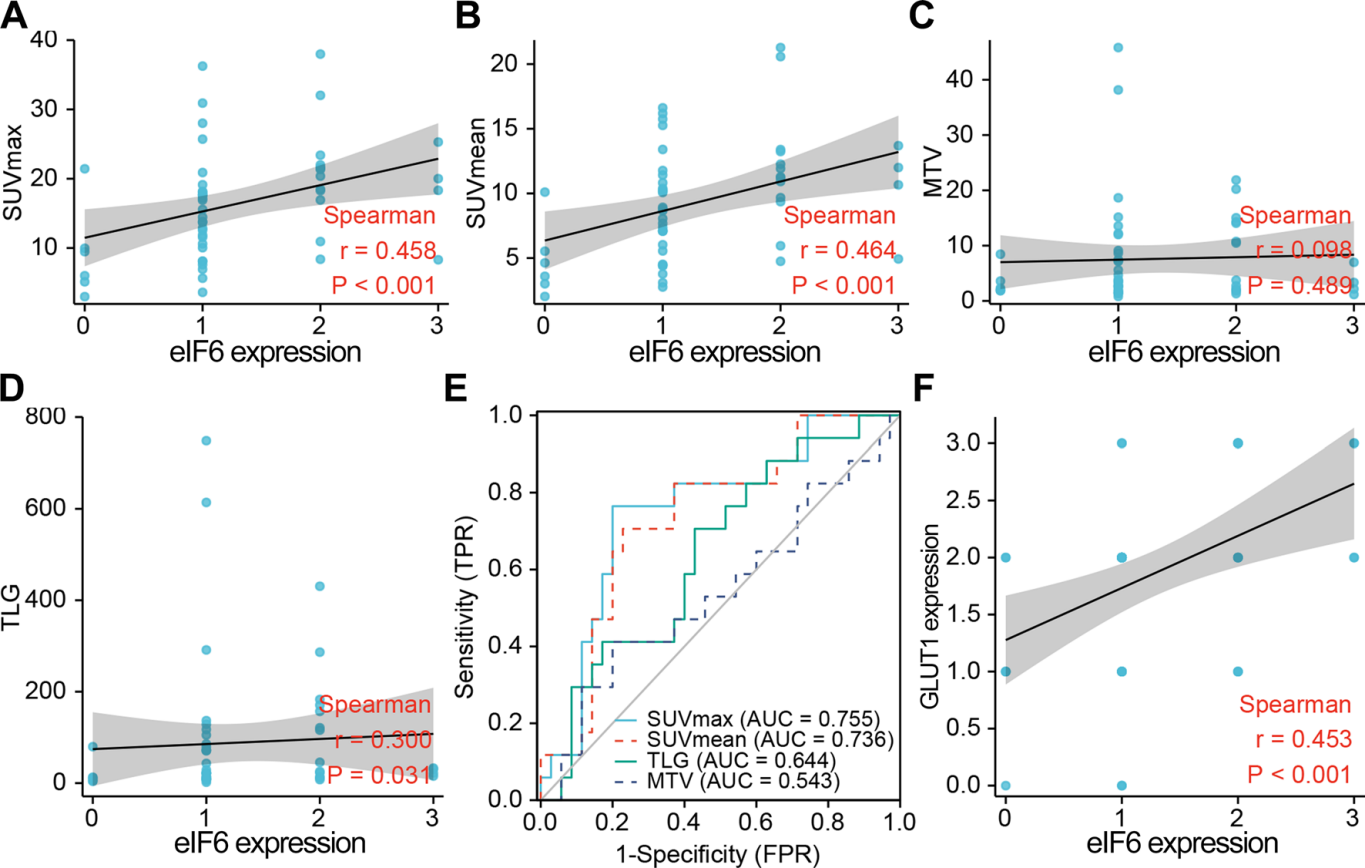
eIF6 is positively correlated with FDG accumulation in ESCA patients. **A** Scatter plots showing the correlation of SUVmax, SUVmean (**B**), MTV (**C**), and TLG (**D**) with the protein level of eIF6. **E** Determination of the cutoff value of SUVmax, SUVmean, TLG, and MTV from the ROC curve showing the prediction performance of eIF6. The area under the SUVmax, SUVmean, TLG and MTV ROC curve was 0.755, 0.736, 0.644, and 0.543, respectively. **F** The expression of eIF6 protein was significantly positively correlated with GLUT1, r = 0.453

To further investigate whether eIF6 influences tumor metabolism in ESCA, ^18^F-FDG PET/CT metabolic parameters were analyzed based on the eIF6 expression in 52 ESCC patients (Table 2). The SUV_max_ and SUV_mean_ were larger in ESCC patients with high eIF6 expression than in the low eIF6 expression group (p < 0.05). There was no significant statistical differences in TLG and MTV according to the eIF6 expression. Representative PET/CT images of ESCA patients with high (Fig. 3A) or low (Fig. 3B) SUV_max_ are displayed. Patients with higher values of the primary lesion SUV_max_ had higher expression of eIF6 and GLUT1, compared to those with lower SUV_max_ values (p < 0.05, Fig. 3C, D). Besides, there was significant correlation between eIF6 protein levels and SUVmax, SUVmean or TLG (rho = 0.458, 0.460, and 0.300, respectively, p < 0.01, Fig. 4A, B, D). However, eIF6 intensity score showed no statistical correlation with MTV (Fig. 4C). These results demonstrated that eIF6 is associated with glucose metabolism in ESCA.

**Table 2:** Comparison of PET metabolic parameter according to eIF6 expression

PET metabolic parameter	eIF6—low (n = 35)	eIF6—high (n = 17)	*p*
SUV_max_ (mean ± SD)	14.46 ± 7.69	20.3 ± 7.45	0.012*
SUV_mean_ (mean ± SD)	8.18 ± 4.06	11.59 ± 4.47	0.008*
TLG (median, range)	21.98 (9.64–79.68)	32.81 (18.59–163.81)	0.095
MTV (median, range)	3.57 (1.9–8.63)	3.74 (2.06–14.18)	0.619

### Predictors of eIF6 expression in ESCA patients

We next sought to determine the threshold of PET parameters that would predict tumor eIF6 status in primary ESCA. ROC curve analysis revealed a SUV_max_ and SUV_mean_ cutoff values of 18.2 and 10.52, respectively, which were related to AUC of 0.755 (sensitivity 76.5%, specificity 80.0%) and 0.736 (sensitivity 70.6%, specificity 77.1%) (Fig. 4E). Next, we analyzed the relationship between eIF6 and GLUT1 expression. The data showed a positive correlation between the immunohistochemistry scores of eIF6 and GLUT1 (rho = 0.453, p < 0.001, Fig. 4F).

### Suppression of eIF6 inhibits the ESCA cell proliferation and migration

**Fig. 5:**
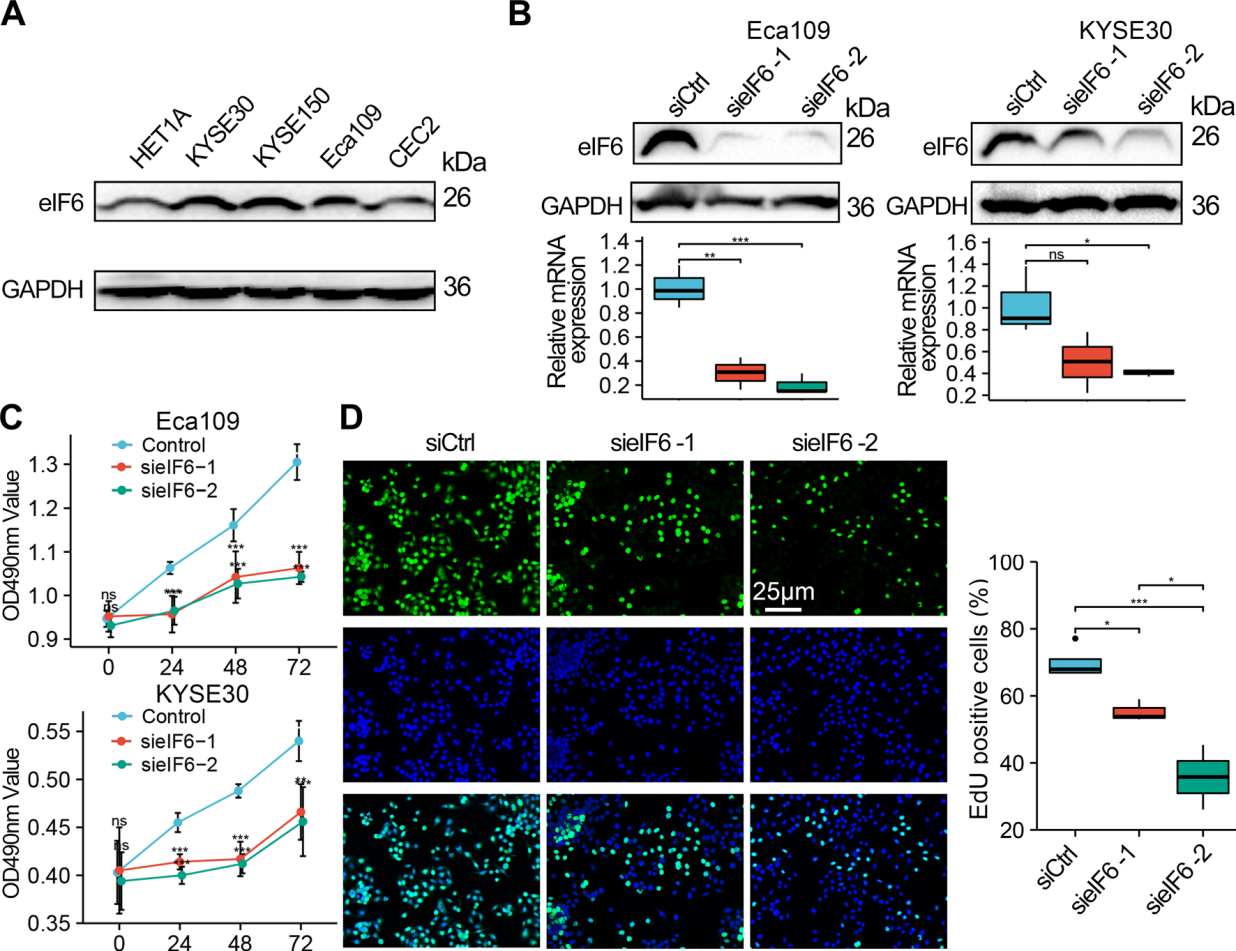
Silencing eIF6 expression inhibited the proliferation of ESCA cells. **A** Western blot analysis of eIF6 protein in normal epithelial cells (HET1A) and different ESCA cell lines. **B** qRT-PCR and Western blot analysis confirming the success of eIF6-siRNAs transfection. **C** Silencing of eIF6 expression reduced the growth of Eca109 and KYSE30 cells as revealed by MTS assays at 24, 48, and 72 h after transfection. **D** Cell proliferation decreased after eIF6-siRNAs treatment as detected by *EdU* staining. *p < 0.05, **p < 0.01, ***p < 0.001

To explore the anti-tumor effect of eIF6 on human ESCA, we evaluated the eIF6 protein expression in four ESCA cell lines and a human epithelial cell line (HET1A). Compared with HET1A, there was higher expression of eIF6 in KYSE30, KYSE150, and Eca109 cells (Fig. 5A). Specific siRNAs were used to silence eIF6 in Eca109 and KYSE30 cells, and interfering efficiency was confirmed by qRTPCR and Western blot analysis (Fig. 5B). We determined cell viability using both MTS assay (Fig. 5C) and *EdU* proliferation assay (Fig. 5D). The results showed that the cell viability of both siRNA (sieIF6) group was significantly lower than that of scramble siRNA control (siCtrl) group (p < 0.05).

**Fig. 6:**
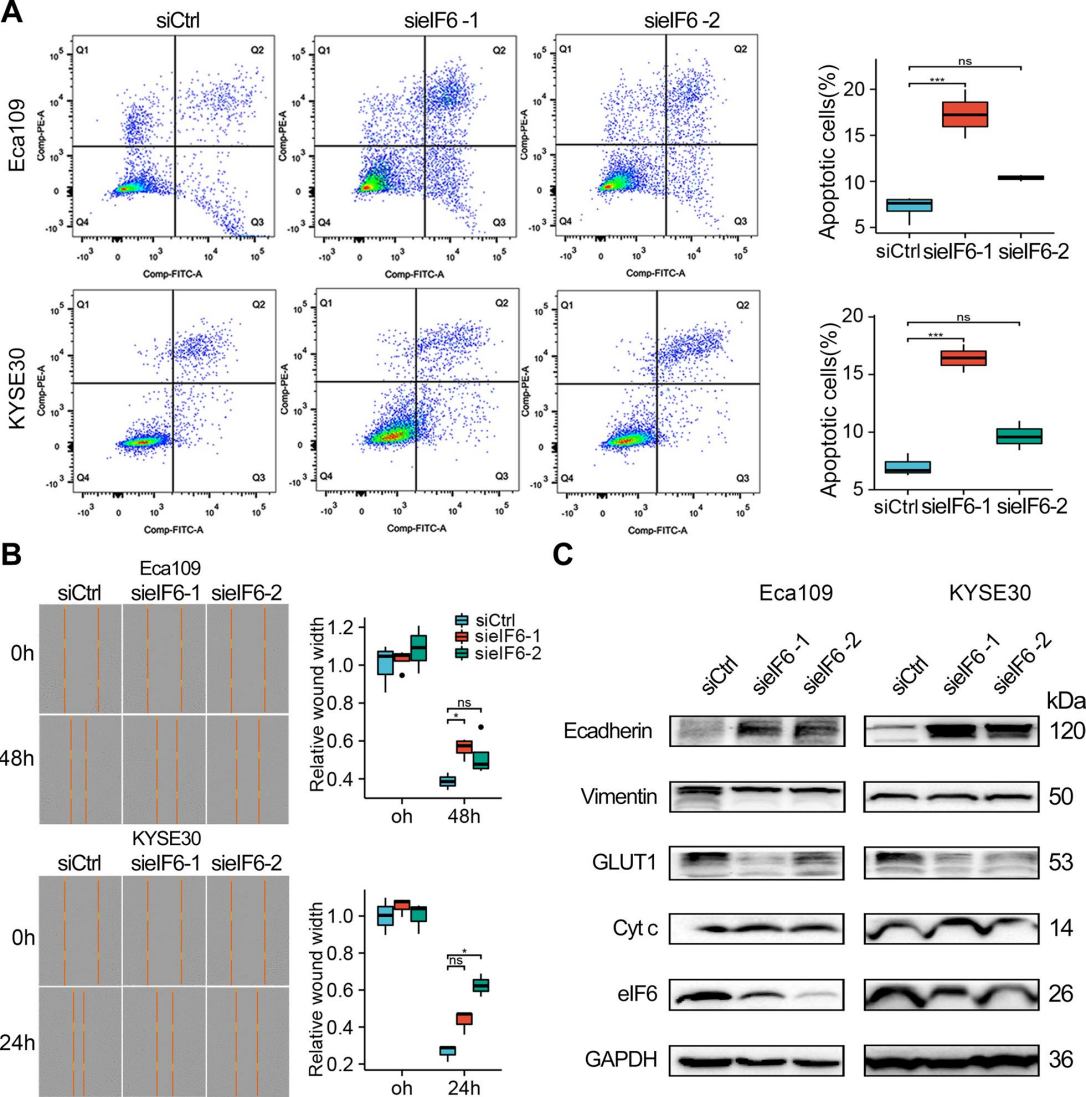
Knockdown eIF6 induced apoptosis and inhibited cell migration in ESCA. **A** The effect of eIF6 knockdown on apoptosis of Eca109 and KYSE30 cells as detected by flow cytometry. **B** Representative images and quantification showing results of the wound healing assay in ESCA cells. **C** Western blotting displayed the protein expression following eIF6 siRNA transfection in Eca109 and KYSE30 cells. *p < 0.05, **p < 0.01, ***p < 0.001

It was evident from the FCM assay that treatment with eIF6 siRNA#1 induced a higher percentage of apoptotic cells as compared to those treated with control siRNA (Fig. 6A). In addition, wound healing assay demonstrated that treatment with eIF6 siRNA significantly inhibited migration of ESCA cell lines (Fig. 6B). To understand the potential mechanisms, we performed Western blot analysis. Unlike the siCtrl results in the Eca109 and KYSE30 cells, the eIF6 knockdown suppressed the expression of GLUT1 and Vimentin, a mesenchymal cytoskeletal marker, but promoted the expression of E-cadherin, an epithelial regulator, and Cyt c, which is a biochemical marker for apoptosis (Fig. 6C). These data suggest that eIF6 promotes ESCA cell proliferation and motility.

### The association between eIF6 and Tumor Immune Infiltration in ESCA

**Fig. 7:**
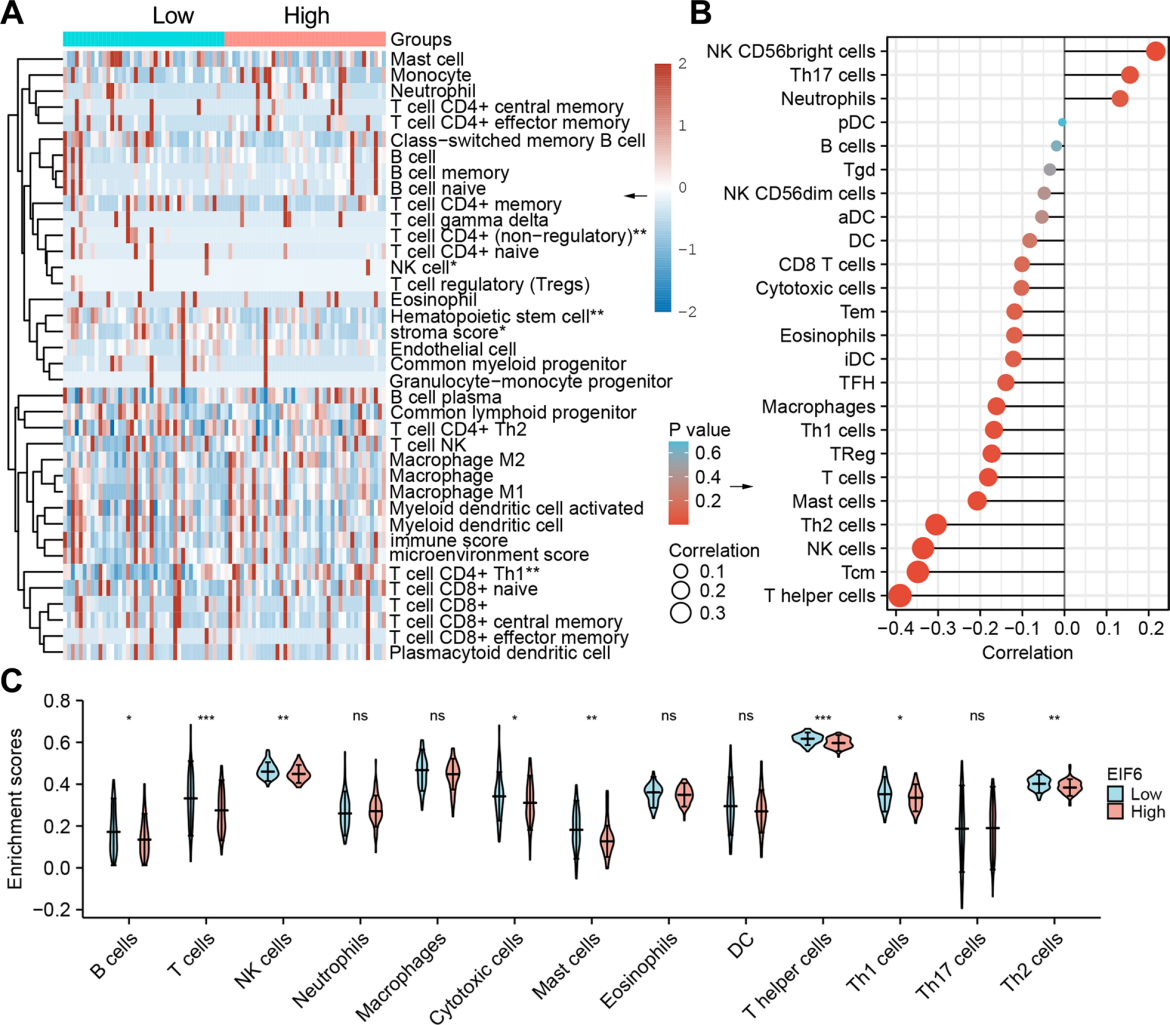
Immune characteristics between eIF6 high and low expression groups. **A** Tumor immune status of the high (n = 81) and low eIF6 expression groups (n = 81) using xCell microenvironment scores in the TCGA datasets. **B** Lollipop plot showing the correlation between the relative abundance of immune cells and eIF6 gene expression levels in the TCGA-ESCA samples determined using the ssGSEA method. **C** Violin plot displaying differences in 13 immune cell types between low and high eIF6 expression groups. *p < 0.05, **p < 0.01, ***p < 0.001

We then assessed whether eIF6 expression was correlated with the characteristics of immune cells in ESCA. The xCell analysis revealed that the abundance of different types of lymphocytes such as, CD4^+^ T cells (non-regulatory), CD4^+^ T cells (Th1), Hematopoietic stem cell, NK cell, and stroma score was statistically different in the eIF6 high group and low group (p < 0 0.05, Fig. 7A). In sync, the lollipop plot of ssGSEA analysis showed that eIF6 expression was negatively correlated with the intensity of immunocytes, such as Tcm (r = − 0.388, p < 0.001), NK cells (r = − 0.386, p < 0.001), T helper cells (r = − 0.368, p < 0.001), Macrophages (r = − 0.208, p = 0.008), T cells, Th2 cells, Th1 cells or iDCs, while positively correlated with the abundance of NK CD56 bright cells (r = 0.232, p = 0.003) and Th17 cells (r = 0.198, p = 0.011) (Fig. 7B). Besides, the violin plot indicated that the eIF6 high expression group had significantly lower B cells, T cells, NK cells, cytotoxic cells, mast cells, T helper cells, Th1 cells, and Th2 cells (p < 0 0.05, Fig. 7C).

### Association between eIF6 expression and immune marker sets in ESCA

**Fig. 8:**
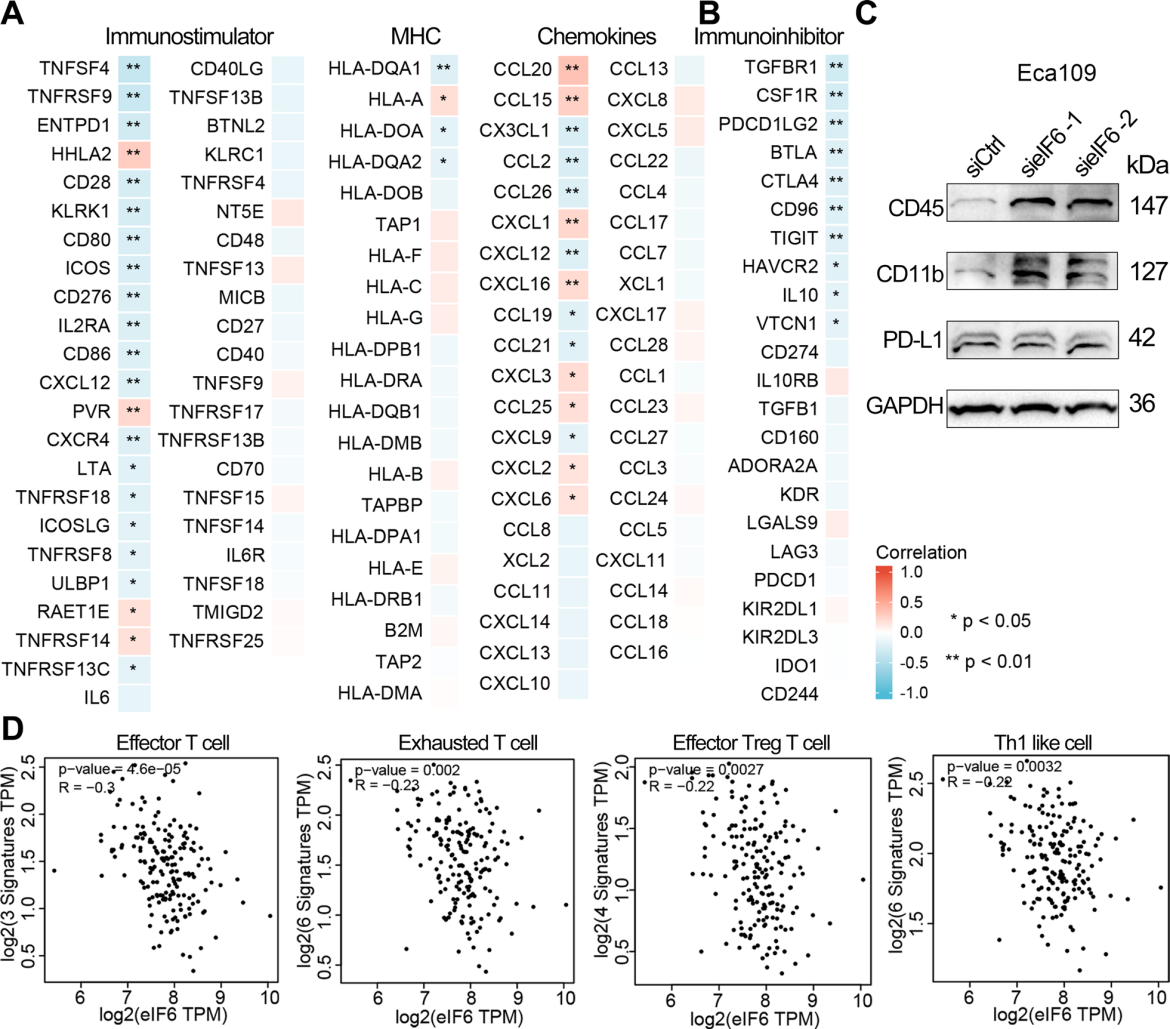
Correlation between eIF6 expression and immune-related genes in human ESCA. **A** A heatmap showing the spearman’s rank correlation between eIF6 expression level and immunostimulators, MHC genes, chemokines, and immunoinhibitors **B** based on the TISIDB database. Positive and negative correlation are indicated by red and blue, respectively. **C** Western blot analysis for CD45, CD11b, and PD-L1 in Eca109 cells with eIF6 knockdown. **D** Scatter plots showing the correlation between immune cells infiltrations and the eIF6 expression using GEPIA2 database. *p < 0.05, **p < 0.01

Further correlation analysis suggested that about half of the immunomodulators (chemokines, MHC-s, and immune stimulators) were negatively correlated with eIF6 in ESCA (Fig. 8A). There were no obvious differences in the correlation regardless of the tumor purity adjustment. Most marker sets of monocytes, TAMs and M2 macrophages exhibited significant correlation with eIF6 expression in both TIMER and GEPIA2 databases (p < 0.05, Table 3). Moreover, CD3E and CD2 of T cells, TBX21 and STAT4 of Th1 phenotype, BCL6, CD278 (ICOS) or CXCL13 of follicular T helper (Tfh) were negatively correlated with eIF6 expression in ESCA (p < 0.05, Table 4). According to the ssGSEA score of the gene sets, there was a negative correlation between eIF6 and various immune checkpoints, such as TGFBR1 (r = − 0.300), CSF1R (r = − 0.274), PD-L2 (PDCD1LG2, r = − 0.238), BTLA, CTLA4, CD96, TIGIT, HAVCR2, IL10, and VTCN1 in ESCA (p < 0.05, Fig. 8B). TIMER and GEPIA2 correlation analyses demonstrated that the immune marker genes of Treg and T cell exhaustion, such as CD25 (IL2RA), CTLA4, LAG3 and TIM-3 (HAVCR2) were significantly correlated with eIF6 expression in ESCA (Table 4). These findings were also observed in the GEPIA2 database. Similarly, eIF6 was negatively correlated with the immune signature of effector T cells (r = − 0.3), exhausted T cells (r = − 0.23), effector Treg T cells (r = − 0.22), and Th1 like cells (r = − 0.22) in ESCA (Fig. 8D). As illustrated in Fig. 8C, the protein level of CD45 and CD11b were enhanced in Eca109 cells after eIF6 knockdown, whereas the PD-L1 expression was not obviously affected as evident by the immunoblot analysis. Taken together, these findings suggested that eIF6 might participate in the immune responses and immune escape within the TME in ESCA.

**Table 3:** Correlation analysis between eIF6 and relate genes and markers of innate immunity cells in ESCA

Description	Genemarkers	TIMER (n = 184)				GEPIA2 (n = 181)	
		Purity		None			
		Cor	p	Cor	p	Cor	p
Monocyte	CD14	− 0.154	*	− 0.132	0.073	− 0.094	0.210
	CD86	− 0.286	*	− 0.262	*	− 0.230	*
	CD16 (FCGR3A)	− 0.238	*	− 0.223	*	− 0.190	*
TAM	CD68	0.139	0.062	0.156	*	0.140	0.061
	CCL2	− 0.266	*	− 0.262	*	− 0.130	0.077
	CCL5	− 0.117	0.116	− 0.125	0.090	− 0.081	0.280
M1 Macrophage	INOS (NOS2)	0.137	0.066	0.129	0.080	0.160	*
	CXCL10	− 0.129	0.083	− 0.121	0.101	− 0.018	0.800
	TNF-α (TNF)	− 0.029	0.695	− 0.008	0.913	− 0.028	0.710
M2 Macrophage	CD206 (MRC1)	− 0.203	*	− 0.200	*	− 0.150	*
	CD163	− 0.208	*	− 0.199	*	− 0.094	0.210
	IL10	− 0.177	*	− 0.182	*	− 0.150	*
Neutrophils	CD66b (CEACAM8)	0.142	0.057	0.113	0.127	0.001	0.980
	CD11b (ITGAM)	− 0.189	*	− 0.166	*	− 0.170	*
	CCR7	− 0.039	0.601	− 0.059	0.427	− 0.180	*
	CD15 (FUT4)	0.085	0.256	0.071	0.340	0.110	0.160
Natural killer cell	KIR2DL1	0.052	0.486	0.041	0.584	− 0.090	0.230
	KIR2DL3	− 0.167	*	− 0.159	*	− 0.091	0.220
	KIR2DL4	0.037	0.625	0.035	0.633	− 0.025	0.740
	KIR3DL1	0.022	0.766	0.013	0.858	− 0.095	0.200
	KIR3DL2	− 0.107	0.153	− 0.107	0.147	− 0.029	0.700
	KIR3DL3	− 0.001	0.993	0.003	0.963	− 0.048	0.520
	KIR2DS4	0.041	0.586	0.053	0.470	− 0.095	0.200
Dendritic cell	HLA-DPB1	− 0.163	*	− 0.165	*	− 0.210	*
	HLA-DQB1	− 0.078	0.294	− 0.093	0.210	− 0.096	0.200
	HLA-DRA	− 0.129	0.083	− 0.136	0.065	− 0.110	0.130
	HLA-DPA1	− 0.137	0.065	− 0.138	0.061	− 0.130	0.070
	BDCA-1 (CD1C)	− 0.250	*	− 0.249	*	− 0.250	*
	BDCA-4 (NRP1)	− 0.213	*	− 0.215	*	− 0.100	0.170
	CD11c (ITGAX)	− 0.178	*	− 0.168	*	− 0.210	*
	NKp46 (NCR1)	− 0.031	0.681	− 0.045	0.547	− 0.100	0.170

TAM, tumor associated macrophage; Cor, R value of Spearman’s correlation. Purity, correlation adjusted by purity. *p < 0.05. None, correlation without adjustment

**Table 4:** Correlation analysis between eIF6 and relate genes and markers of adaptive immunity cells in ESCA

Description	Genemarkers	TIMER (n = 184)				GEPIA2 (n = 181)	
		Purity		None			
		Cor	p	Cor	p	Cor	p
CD8 + Tcell	CD8A	− 0.136	0.067	− 0.144	0.050	− 0.130	0.086
	CD8B	− 0.070	0.347	− 0.087	0.241	− 0.110	0.140
T cell	CD3D	− 0.108	0.147	− 0.114	0.121	− 0.170	*
(general)	CD3E	− 0.143	0.055	− 0.155	*	− 0.200	*
	CD2	− 0.167	*	− 0.178	*	− 0.190	*
Bcell	CD19	− 0.009	0.906	− 0.026	0.728	− 0.120	0.120
	CD20 (MS4A1)	− 0.051	0.496	− 0.070	0.345	− 0.100	0.160
	CD138 (SDC1)	− 0.084	0.262	− 0.054	0.466	− 0.044	0.550
	CD23 (FCER2)	− 0.004	0.960	− 0.019	0.792	− 0.110	0.130
Th1	T-bet (TBX21)	− 0.162	*	− 0.172	*	− 0.190	*
	STAT4	− 0.242	*	− 0.242	*	− 0.200	*
	STAT1	− 0.097	0.195	− 0.089	0.229	0.003	0.960
	IFN-γ (IFNG)	− 0.094	0.207	− 0.090	0.222	− 0.150	*
	TNF-α (TNF)	− 0.029	0.695	− 0.008	0.913	− 0.028	0.710
Th2	GATA3	− 0.124	0.097	− 0.117	0.113	− 0.092	0.220
	STAT6	− 0.038	0.612	− 0.037	0.616	0.057	0.440
	STAT5A	− 0.072	0.334	− 0.081	0.276	− 0.063	0.390
	IL13	− 0.116	0.119	− 0.112	0.130	− 0.140	0.061
Tfh	BCL6	− 0.382	*	− 0.348	*	− 0.270	*
	IL21	− 0.112	0.132	− 0.106	0.152	− 0.180	*
	CD278 (ICOS)	− 0.225	*	− 0.215	*	− 0.190	*
	CXCL13	− 0.169	*	− 0.174	*	− 0.170	*
Th17	STAT3	− 0.085	0.257	− 0.073	0.321	− 0.042	0.570
	IL17A	0.226	*	0.205	*	0.053	0.480
Treg	FOXP3	− 0.245	*	− 0.239	*	− 0.210	*
	CCR8	− 0.260	*	− 0.266	*	− 0.230	*
	STAT5B	− 0.166	*	− 0.160	*	− 0.084	0.260
	TGFβ (TGFB1)	− 0.122	0.103	− 0.091	0.217	− 0.076	0.310
	CD25 (IL2RA)	− 0.255	*	− 0.248	*	− 0.180	*
T cell exhaustion	PD-1 (PDCD1)	− 0.116	0.121	− 0.120	0.104	− 0.130	0.078
	CTLA4	− 0.205	*	− 0.205	*	− 0.200	*
	LAG3	− 0.158	*	− 0.157	*	− 0.085	0.250
	TIM-3 (HAVCR2)	− 0.250	*	− 0.236	*	− 0.230	*
	GZMB	− 0.108	0.149	− 0.108	0.144	− 0.099	0.180

TAM, tumor associated macrophage; Cor, R value of Spearman’s correlation. Purity, correlation adjusted by purity. *P < 0.05. None, correlation without adjustment

## Discussion

The eukaryotic translation initiation factor eIF6 plays an essential role in cell growth and transformation, apoptosis, mitochondrial respiration, as well as lipogenic and glycolytic process [15, 21, 22, 36]. Although overexpression and oncogenic functions of eIF6 have been documented in other cancers, the role and biological functions of eIF6 in ESCA remains poorly understood. Herein, our results demonstrated that eIF6 was up regulated both in ESCA tissues and cell lines, and high eIF6 expression led to poor prognosis in EA patients, with a favorable diagnostic reference value in ESCA. Moreover, knockdown of eIF6 significantly suppressed cell proliferation and migration, and induced cell apoptosis in the ESCA cells. The PET parameters have previously shown a remarkable potential for predicting gene expression status in cancers [37–39]. Our IHC analysis revealed that tumor eIF6 expression was positively correlated with FDG PET parameters in ESCA tissues. In addition, SUV_max_ and SUV_mean_ might act as suitable predictors of eIF6 expression in patients with ESCA. On the other hand, the bioinformatics analyses suggested that eIF6 is involved in metabolic pathways and tumor immune infiltration in ESCA. These findings emphasize the oncogenic role of eIF6 and its therapeutic potential in the ESCA.

Our bioinformatics analyses demonstrated that high eIF6 expression predicted worse prognosis in ESCA patients. This was in sync with observations for human colorectal cancer, biliary tract cancer, hepatocellular carcinoma and lung adenocarcinoma [17, 19, 20, 40]. A previous study showed that eIF6 knockout mice impacted Myc-induced lymphomagenesis and tumor progression by modulating p53 [15]. Another study showed that high eIF6 expression is significantly associated with clinicopathological features, such as lymph node metastases in ovarian serous carcinoma [18]. Through IHC staining analysis, Gantenbein et al. [17] demonstrated significant differences in the eIF6 expression between higher and lower grade lung adenocarcinoma, but not in lung squamous cell carcinoma. In our study, eIF6 knockdown in ESCA cells inhibited cell migration via regulation of EMT marker genes. However, our data showed that eIF6 expression was not significantly associated with lymph node metastasis and p stages in ESCA tumors, probably due to different tumor-specific roles of eIF6 or small sample size.

Previously, Scagliola et al. [22] postulated that eIF6 depletion delays the liver disease progression from non-alcoholic fatty liver disease to hepatocellular carcinoma in vivo. Mechanistically, eIF6 depletion regulated mitochondrial respiration by targeting the mTORC1-eIF4F-YY1 translational axons. Cancer cells preferentially use aerobic glycolysis for stimulate ATP generation and lactate production (Warburg effect) [41]. Interestingly, inhibition of eIF6 could reduce cell growth by impairing lactate and ATP production in Malignant Pleural Mesothelioma [42]. Moreover, a previous systematic review demonstrated that eIF6 promoted glycolytic flux and fatty acid synthesis and increased tumor viability. But the mechanism through which mTOR or Myc regulate the activity of eIF6 need to be further investigated [43]. Our GSEA results demonstrated that the highly expressed eIF6 in ESCA patients regulated several metabolic pathways such as HALLMARK_MYC_TARGETS, HALLMARK_OXIDATIVE_PHOSPHORYLATION, REACTOME_GLYCOLYSIS, and KEGG_GLYCOLYSIS_GKYCIBEIGENES. In recent years, ^18^F-FDG PET/CT has emerged as a noninvasive diagnostic tool for evaluating tumor glycolytic activity, diagnosing and staging of various malignant tumors [44, 45]. Molecular imaging can be applied to reveal the molecular profile of cancers [38, 46, 47]. This study found that high eIF6 expression was positively correlated with FDG uptake (in terms of SUV_max_ and SUV_mean_) and the expression of GLUT1 in ESCA tissue. These results are in agreement with data from previous studies [12]. In addition, it was found that the SUV_max_ cutoff for PET/CT parameter was 18.2, which was more effective compared to the SUV_mean_, TLG, and MTV in predicting eIF6 expression. For these reasons, noninvasive methods, such as molecular imaging, could be used for predicting eIF6 status have great clinical relevance.

Evidence from previous studies has shown that immune cells or immune-related biomarkers in the TME can predict the survival outcomes and influence response to immune checkpoint therapy [48, 49]. By combining contrast-enhanced CT images and RNA-seq genomic data from tumour biopsies, Roger et al. demonstrated that imaging biomarker could be useful in estimating CD8 cell count and predicting clinical outcomes of patients treated with anti-PD-1 and PD-L1[49]. The dual roles of mutation burden and MS-indels was identified in predicting outcomes of central nervous system and synchronous cancers following immune checkpoint inhibitors (ICIs) treatment [50]. Thus, analysis of the cancer-specific immune biomarkers may reveal novel molecular targets for ESCA treatment. Here, using the xCell algorithm analysis, eIF6 was found to be negatively correlated with CD4 + T cells, hematopoietic stem cell, NK cell, T helper cells, macrophages, T cells, Th2 cells, Th1 cells, iDCs, among others. Moreover, the expression of eIF6 were negatively correlated with immune marker genes, such as genes of monocytes, TAMs, M2 macrophages, T cell, Th1, and Tfh phenotype. These result demonstrate that eIF6 expression may be negatively correlated with immune cells including macrophages, T cells, and Th1 cells. We subsequently verified that showed that eIF6 silencing increased the expression of macrophage markers CD45 and CD11b in ESCA cells. Previous studies suggested that cancer cells also acquired immune regulatory membrane proteins such as PD-L1, CD4, CD45, CTLA4 and Tim3 expressed in lymphocytes, which in turn contribute to the development of the immunosuppressive tumor microenvironment [51–53]. Whether CD45 or CD11b -positive cancer cells could regulate the ESCA tumor microenvironment remains further exploration. Consistently, a previous study revealed that eIF6 overexpression increase the number of activated T cells [16, 25]. Moreover, eIF6 overexpression induced the metabolic switch in CD4^+^ T cells [26], and was negatively correlated with most immunomodulators (chemokines, MHC-s, immune stimulators) in ESCA. Similarly, we found a negative correlation between eIF6 expression and immune checkpoints, including CTLA4, and HAVCR2, in ESCA. It has been reported that CTLA4 activates CD4 + and CD8 + T cells, whereas TIM-3 functions as a negative regulator of T cell activation and also involved in exhaustion, Th1 responses [54, 55]. Inhibitors of CTLA4 and TIM-3 can be applied as immunotherapeutic targets in the treatment of cancer patients [56, 57]. This suggests that eIF6 might serve as a potential biomarker of immune cell infiltration in ESCA.

There are several limitations to this study. First, although we found that high expression of eIF6 was associated with poor prognosis of ESCA patients, this finding was not experimentally validated using clinical samples. Second, although metabolic and genomic signatures were combined to investigate potential biomarkers and mechanisms, more samples and additional validation studies were needed. Third, further in-depth clinical research should be conducted to clarify the functional role of eIF6 in the tumor immunosuppressive microenvironment.

## Conclusions

In conclusion, this study shows that eIF6 is highly expressed in ESCA tumor tissues and could predicted worse prognosis. Regarding biological functions, we demonstrated that eIF6 expression influenced preoperative FDG uptake and involved in immune cell infiltration in ESCA, which provides novel insights to the tumor biology. eIF6 is likely to be prognostic biomarker for ESCA. Further prospective experiments should be carried out to verify the expression and function of eIF6 in ESCA.

## Abbreviations

eIF6: Eukaryotic initiation factor 6
TCGA: The Cancer Genome Atlas
ESCA: Esophageal carcinoma
EA: Esophageal adenocarcinoma
ESCC: Esophageal squamous cell carcinoma
IHC: Immunohistochemical
GLUT1: Glucose transporter-1
^18^F-FDG-PET: 18F-fuorodeoxyglucose positron emission tomography
CT: Computed tomography
SUV_max_: Maximum standardized uptake value
ROI: Region of interest
MTV: Metabolic tumor volume
TLG: Total lesion glycolysis
OS: Overall survival
ROC: Receiver operating characteristic
AUC: Area under curve
HET1A: Human epithelial cell line
NPM1: Nucleophosmin 1
EIF2S2: Eukaryotic translation initiation factor 2 Subunit β
METTL3: Methyltransferase 3
TME: Tumor microenvironment
PD-1: Programmed death-1
PD-L1: Programmed death ligand-1
CTLA4: Cytotoxic T lymphocyte associated antigen 4
Cyt c: Cytochrome c
GO: Gene ontology
KEGG: Kyoto Encyclopedia of Genes and Genomes
GSEA: Gene Set Enrichment Analysis
PPI: Protein–protein interaction
STRING: Search Tool for the Retrieval of Interacting Genes/Proteins
TIMER: The Tumor Immune Estimation Resource
GSVA: Gene set variation analysis
TISIDB: Tumor Immune System Interactions
siRNA: Small interfering RNA
